# Total mercury and methylmercury (MeHg) in braised and crude *Boletus edulis* carpophores during various developmental stages

**DOI:** 10.1007/s11356-021-15884-1

**Published:** 2021-08-12

**Authors:** Jerzy Falandysz, Martyna Saba, Małgorzata Rutkowska, Piotr Konieczka

**Affiliations:** 1 Department of Toxicology, Faculty of Pharmacy, Medical University of Lodz, 1 Muszyńskiego Street, 90-151 Łódź, Poland; 2 Laboratory of Environmental Chemistry and Ecotoxicology, Faculty of Chemistry, University of Gdańsk, 63 Wita Stwosza Street, 80-308 Gdańsk, Poland; 3 Department of Analytical Chemistry, Faculty of Chemistry, Gdańsk University of Technology, 11/12 G. Narutowicza Street, 80-233 Gdańsk, Poland

**Keywords:** Culinary processing, Food toxicology, Forest, Fungi, Mushrooms, Wild food

## Abstract

We collected and processed *Boletus edulis* (King Bolete) carpophores grouped in four batches based on their developmental stage (button stage, young—white, large—white, and large—yellow). The study aimed, for the first time, to examine the *B. edulis* content and effect of braising and to estimate the intake of total mercury (THg) and methylmercury (MeHg) from a single meal based on whole (wet) weight (ww) and dry weight (dw). In braised carpophores, THg concentrations ranged from 0.2668 ± 0.0090 to 0.5434 ± 0.0071 mg kg^−1^ ww at different developmental stages, whereas crude products concentrations ranged from 0.1880 ± 0.0247 to 0.2929 ± 0.0030 mg kg^−1^ ww. The button stage crude carpophores were more highly contaminated with THg than at later stages of maturity, but MeHg levels were lower (*p* < 0.0001). On the other hand, braised button stage carpophores showed more MeHg than at later maturity stages. MeHg contributed at 1.9 ± 0.7% in THg in crude mushrooms and at 1.4 ± 0.3% in braised meals. The effect of braising was to increase the average THg and MeHg contents in fresh mushroom meals by 52 ± 31% and 53 ± 122% respectively, but a reduction of 40 ±14% and 40 ± 49% respectively was seen on a dw basis. The potential intakes of THg and MeHg from braised meals of *B. edulis* studied were small and considered safe.

**Graphical abstract:**

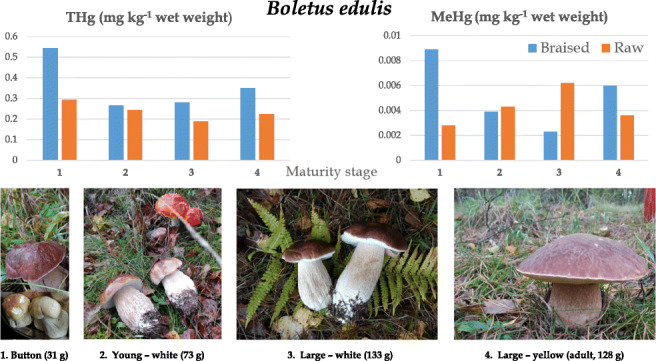

## Introduction

*Boletus edulis* Fr. is a prized edible wild mushroom with many common names in different languages, including, Borowik szlachetny (boletus noble), Cep, Cèpes, King Bolete, Porcini, Prawdziwek (true mushroom), Steinpilz (stone mushroom), Stensopp (stone mushroom), Penny Bun, and more. This diversity of names reflects the geographical extent of the appreciation and value of this food which is a target of foragers as well as recreational mushroom pickers. *B. edulis* and the *Lactarius deliciosus* (L.) Gray (tasty in Latin) are among the mushrooms mentioned by Adam Mickiewicz, the National Guard of the time, in a colorful description of the mushrooming habit in the Polish national epic poem, the “Pan Tadeusz,” published in 1834.

**Fig. 1:**
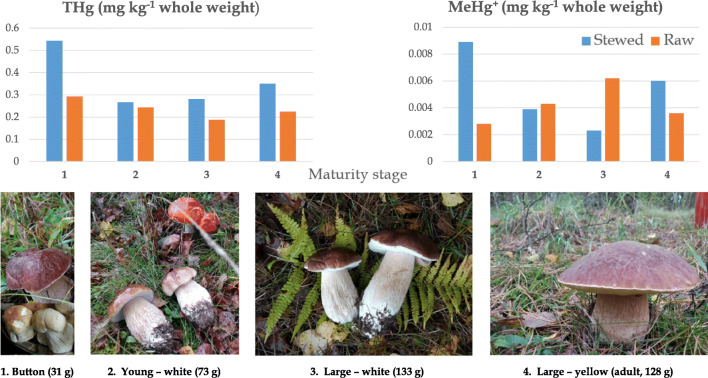
Graphical representation of the stages of maturity of the *B. edulis* tested and of THg and MeHg data in products

The King Bolete forms relatively easily recognizable carpophores (fruiting bodies; Fig. 1). It forms mycorrhizal relationships with conifers, primarily spruce *Picea abies* (L.) H.Karst, pine *Pinus sylvestris* L., and birch *Betula* spp. (Gumińska and Wojewoda 1985). Carpophores of *B. edulis* are considered highly nutritious and, with a moisture content of 90% in fresh specimens (Table 1) as seen in the study by Stijve and Roschnik (1974). A recent study (Heleno et al. 2015) has characterized a number of nutrients and other organics in *B. edulis*:

- Carbohydrates (mostly in the form of undigestible chitin, which has a fiber-like role in human bowel function) in dried fruiting bodies, accounted for 81.86 ± 0.41%
- Proteins at 10.65 ± 0.47%
- Fatty acids (polyunsaturated predominate over mono- and saturated) including palmitic, stearic, oleic, linoleic, and alfa linolenic acid and eighteen other less abundant fatty acids (total fat content was at 2.23 ± 0.02%)
- Free sugars such as fructose at 0.71 ± 0.33%, glucose at 1.24 ± 0.46%, mannitol at 3.14 ± 1.18, and trehalose at 9.29 ± 0.51%
- Tocopherols such as α-tocopherol and β-tocopherol
- Organic acids such as oxalic acid and fumaric acid (no quinic, malic, or citric acids)
- And phenolic compounds such as protocatechuic acid, *p*-hydroxybenzoic acid *p*-coumaric acid

**Table 1:** Summary of data on the samples size, biomass (whole weight; ww), and moisture content of the fungal materials

Developmental stage of carpophore and number of the specimens	Stewed carpophores		Crude (control) carpophores	
	Biomass (g; ww)	Moisture content (%)	Biomass (g; ww)	Moisture content (%)
Button stage (*n* = 19)	233.7	75.5	231.0	90.0
Young—white (*n* = 8)	242.4	74.3	245.5	90.0
Large—white (*n* = 3)	185.8	76.5	190.6	90.1
Large—yellow (*n* = 4)	241.8	73.1	238.2	90.1
Mean	225.9 ± 23.4	74.8 ± 1.3	226.3 ± 21.2	90.0 ± 0.0

The antioxidant properties of methanol extracts from dried *B. edulis* relating to phenolic compounds and tocopherols were associated largely with radicals scavenging activity and reducing power and less to lipid peroxidation or β-carotene bleaching inhibition (Heleno et al. 2015).

*B. edulis* is also relatively rich in important inorganic constituents and particularly in selenium (Se), occurring in the range from 4.1 to 63 mg kg^−1^ dry weight (mean weighted content is at around 20 mg kg^−1^ dw) in specimens foraged from European and North American habitats (Falandysz 2013). Mean Se in the caps of *B. edulis* from Poland was in the range 8.7 ± 8.9 to 32 ± 20 mg kg^−1^ dw (Falandysz et al. 2008). The zinc (Zn) content in caps ranges from 120 ± 30 to 210 ± 60 mg kg^−1^ dw, copper (Cu) from 25 ± 11 to 64 ± 27 mg kg^−1^ dw, and iron (Fe) from 34 ± 10 to 110 ± 90 mg kg^−1^ dw. Stems have relatively lower contents than the caps—around twice less in Zn, ~ 2 to 3-fold less in Cu and up to 2-fold lower in Fe (Falandysz et al., 2008 and 2011; Frankowska et al. 2010). *B. edulis* effectively bio-concentrates minerals as Cu, Zn, potassium (K), and magnesium (Mg) in its carpophores (Falandysz et al. 2011). It bio-excludes lead (Pb) and contamination levels are typically below 2 mg kg^−1^ dw (Falandysz et al. 2008; Širić et al. 2017), but it strongly bio-concentrates Cd and typical contamination levels are up to 10 mg kg^−1^ dw in caps (Falandysz et al. 2011; Širić et al. 2017).

Mercury is naturally dispersed in trace amount in the Earth’s crust and soils may be regionally enriched in this element due to specific geological history (Gustin et al. 1999). Forest ecosystems play a key role in biogeochemical cycling of Hg (Falandysz 2017; Zhou et al. 2020). It has been documented in studies that forest soil is a source of Hg for a diversity of macrofungi, which is found to be relatively resistant to accumulation in the sclerotia (a dense mass of mycelia that is buried underground) formed by some wood decaying subtropical fungi such as *Wolfiporia cocos* (F.A. Wolf) Ryvarden and Gilb., or *Pleurortus tuber-regium* (Rumph. ex Fr.) Singer (Alonso et al. 2000; Árvay et al. 2014; Bargagli and Baldi 1984; Falandysz et al. 2001, 2002, 2013, 2020b; Jarzyńska et al. 2012; Kavčič et al. 2019; Kojta et al. 2015; Krasińska and Falandysz 2016; Melgar et al. 2009; Nasr and Arp 2011; Nnorom et al. 2013; Rieder et al. 2011; Saba et al. 2016; Širić and Falandysz 2020; Wiejak et al. 2016).

Wild mushrooms accumulate the largest amount of Hg in the terrestrial environment. Mercury has been found at up to 22 mg kg^−1^ dw in caps and in 8.4 mg kg^−1^ dw in stems of *Boletus bainiugan* Dentinger, from anthropogenically unpolluted region of Yunnan (China), which is a land with largely geogenic Hg in forest topsoils with concentrations of 3.4 mg kg^−1^ dw (Falandysz et al. 2015). Mushrooms collected from former cinnabar (HgS) mining and smelting areas in Slovakia showed a maximal Hg concentration up to 470 mg kg^−1^ dw in *Lactarius quietus* (Fr.) Fr., (Árvay et al. 2014). In the case of *B. edulis,* concentrations up to 100 ± 8 mg kg^−1^ dw have been reported in young carpophores from the HgS mining area in Slovenia (Kavčič et al. 2019). *B. edulis* has a high potential to bioconcentrate Hg and “typical” contamination levels in specimens from European specimens were in the range 1.2 ± 1.4 to 7.6 ± 3.1 mg kg^−1^ dw in caps and from 0.84 ± 0.74 to 3.8 ± 1.5 mg kg^−1^ dw in stems (Falandysz et al. 2003 and 2007; Gucia and Falandysz 2003; Kojta and Falandysz 2016; Melgar et al. 2009; Širić et al. 2017). In Canada, Hg in the whole carpophores of *B. edulis* has been determined at 2.8 ± 1.6 mg kg^−1^ dw (total range 0.46–5.8 mg kg^−1^ dw) (Nasr et al. 2012).

Methylmercury (MeHg) contributed up to 16.1 % in total Hg (THg) in carpophores of several species of edible and inedible fungi studied in Europe, Asia (China), and North America (USA) (Bargagli and Baldi 1984; Fischer et al. 1995; Rieder et al. 2011; Rutkowska et al. 2021; Stijve and Roschnik 1974). In *B. edulis* the contribution of MeHg to THg in carpophores investigated thus far was 14% (THg concentration of 1.9 mg kg^−1^ dw) (Bargagli and Baldi 1984). Kavčič et al. did not detect MeHg at concentration exceeding its contribution at above 5% in a set of *B. edulis* samples from the unpolluted and polluted (cinnabar mine) sites in Slovenia.

MeHg is of concern because of its persistence, strong bioavailability in foods and high toxicity and is therefore of particular health and environmental relevance. In the environment, it is biosynthesized from Hg(II) in minute amounts by some anaerobic methylating bacteria (sulfate-reducing species) in anoxic condition in sediment, water columns and suspended particles, and soil (Schwesig et al. 1999; Xu et al. 2019). MeHg can possibly also be produced by microaerophilic nitrite-oxidizing bacteria *Nitrospina* in oxic condition in subsurface oceanic waters (Villar et al. 2020).

Thus, Hg in low concentrations is a notorious contaminant of both, vegetarian and nonvegetarian diets. Within nonvegetarian foods, it occurs at lowest levels in farmed livestock and at higher levels in game, but the highest focus is on its presence in fish and seafood (Le Croizier et al. 2020; Nawrocka et al. 2020; Petrova et al. 2020). The exposure to Hg and its effects in human are largely linked to the occurrence of MeHg in the muscle tissues of marine fish and other seafood, where it is a major or almost the sole Hg compound (Brambilla et al. 2013).

MeHg undergoes a pronounced biomagnification in aquatic environment food webs. A tragic history of contamination with MeHg of seafood in the Minamata Bay, which in the period from 1951 to 1968 become heavy polluted with MeHg and Hg(II) discharged in industrial sludge, highlighted the neurotoxic nature of MeHg in exposed humans and cats, in the early 1950s (Harada 1995; Falandysz et al. 2020a). A major target for absorbed MeHg in humans is selenium (Se) in selenoenzymes (e.g., thioredoxin reductases 1 and 2, glutathione peroxidase 4), which otherwise prevents and reverses oxidative damage in the brain (Ralston and Raymond 2018; Spiller 2018). Thus, the co-occurrence of Hg and MeHg with Se and its forms in foods or in a wider sense in any environmental material is important when considering intake rates and possible effect in exposed individuals.

*B. edulis* is considered as an excellent ingredient in many recipes, where fresh carpophores are braised, sautéed, or fried, while button stage and young specimens with firm and crunchy flesh are especially valued for pickling. Large size and older specimens are considered more suitable for drying as they are already partially dehydrated. After removing the pore layer, *B. edulis* is also considered good for preserving dry and its flavor can be enhanced when reconstituted by soaking (15 min) in warm water, squeezed dry, and pureed. The water used for soaking (macerate) should to be reserved according to a traditional recipe and used, which can raise a dilemma as water extracts may contain an excess of radiocaesium (^134/137^Cs) depending on the location of collection (Falandysz et al. 2021a; Saba and Falandysz 2021). Dried mushrooms can be consumed whole when rehydrated or they can be also powdered and soaked for use in a crème soup or sauce.

The culinary processing of mushrooms including household or industrial treatment has a basic effect on their sensory and nutritional features, but the amount of information on the impact of processing on content and forms of mineral constituents in mushroom products and meals is limited. This is despite the high biodiversity of wild species that are consumed reaching around 2000 for edibles worldwide, but also despite the tens of dozens of processing recipes, both traditional and modern (Drewnowska et al. 2017). As already mentioned, there is only a handful of literature on the speciation of Hg in mushrooms and similarly on the effects of culinary processing, i.e., blanching or blanching and pickling on fate of THg, and on occurrence in fried mushrooms (Falandysz and Drewnowska 2015 and 2017; Falandysz et al. 2019a, 2019b; Svoboda et al. 2002). There is no previous data on the impact of a culinary treatment on the fate of MeHg in mushrooms or the impact of braising on the fate of both, MeHg and THg, and on possible human exposure. The objective of this study was to investigate and present data on the effects of braising on THg and MeHg content and fate in *B. edulis* mushrooms at various developmental stages and also assesses dietary intake in view of existing recommendations on safe exposure.

## Materials and methods

Samples of the carpophores of *B. edulis* were collected from an unpolluted forested area (near the villages of Pomlewo and Szklana Góra) at ca 30–50 km southwest of the cities of Gdańsk, Sopot, and Gdynia (Gdańsk coordinates: 54°13′7″N 18°21′28″E). The sampling points were in areas predominated with Downy birch *Betula pubescens* Ehrh., and from mixed areas of the Common oak *Quercus robur* L., and Scots pine *Pinus sylvestris* L., with incursions of Downy birch and Norway spruce *Picea abies* (L.) H. Karst (Falandysz et al. 2021b).

The nearest potential area of contamination to the sampling site in the past is a nonferrous smelter situated ca 27 km southeast of the forest and with a largely unreported history of production (Falandysz 2016). However, as the dominating wind direction is westerly with a proportion from the south during the summer months, the impact of the smelter or the nearby city of Gdańsk is likely to be negligible if at all.

All carpophores were in excellent body condition and were collected within a few hours in the early morning of October, 2019. They were cleaned on site from any substrate debris with a ceramic knife. The maturity was assessed visually and each specimen was assigned to and pooled in one of four groups depending on the developmental stage (Fig. 1) and weighed (Table 1). Group “1” consisted of carpophores at the button stage and contained nineteen individuals in the pool (average biomass 31 g wet weight per specimen), group “2” contained eight young specimens with white hymenophore (average biomass 73 g wet weight per specimen), group “3” contained three large specimens with white hymenophore (average biomass 133 g wet weight per specimen), and group “4” included four large (mature—spreading spores) individuals with yellow hymenophore (average biomass 128 g wet weight per specimen) (Graphical abstract, Table 1).

Each carpophore was split vertically, using a ceramic knife, into two parts that were further grouped accordingly (Table 1) and processed as pooled (composite) samples (Falandysz et al. 2021b). Altogether two pooled samples per group were created in parallel giving a total of eight pooled samples. One set of halves from each developmental stage group was used for the braising experiments and the other set served as a control material for calculations and as a reference to the effects of braising on a whole weight basis.

The set of control samples were chopped into small pieces and dried at 65 °C for 24 h using plastic vegetable drying trays and a commercial dryer, then ground to a fine powder using a blender with ceramic blades and plastic container. Depending on the development stage group, the biomass of this set of carpophores ranged from 190.6 to 245.5 g for the whole weight, corresponding to 19.2 to 24.5 g for the dried samples.

The moisture content of the pooled control materials was determined gravimetrically from the difference in biomass weight for fresh and dehydrated materials. The pulverized fungal materials were transferred to tightly sealed plastic bottles (Wide-Mouth Opaque Amber HDPE Packaging Bottles with Caps, Thermo Scientific™ Nalgene™) and stored under dry and clean conditions at room temperature until further analyses.

The halves of carpophores subjected for braising were chopped into pieces of circa 3 × 3 cm within each pool. Each pool of chopped mushrooms was placed in a ceramic coated metal pot (capacity 1 L) with a glass lid with an aperture. The pots were placed on inductive cookers and gently braised with a portion of fat (a mix of butter and olive oil; 1:1; Falandysz et al. 2021b) for 30 min from the time when the contents reached “boiling” point. These freshly braised mushroom meals were transferred, using a ceramic spoon, into tared plastic containers and weighed. Next, the braised products were deep frozen at −24 °C and lyophilised for 72 h (Labconco Freeze Dry System, Kansas City, MO, USA). The moisture content of the braised fungal materials was determined gravimetrically from the difference in biomass weight between the fresh and lyophilised products. The dehydrated products were ground into a powder using a blender with ceramic blades and plastic container and stored in tightly closed plastic bottles (Wide-Mouth Opaque Amber HDPE Packaging Bottles with Closure, Thermo Scientific™ Nalgene™) under dry and clean conditions at room temperature until analysis. The contents of Hg and MeHg were determined in fully dehydrated materials and calculated both on a dry and whole (wet) weight basis.

Data for the braised products were not corrected for added and sorbed by mushrooms fat content, which may provide, to some extent, a “diluting” effect on the content of Hg and MeHg in the mushroom meals obtained. In addition to fat, traditional recipes for braising mushrooms usually require water, table salt and also cream, onion and black pepper or other spices. However, for these experiments, apart from butter and olive oil, nothing else was added to the stew.

The THg and MeHg contents of the fungal materials in this study were determined using a modification of the method by Maggi et al. (2009). It relays on atomic absorption spectroscopy after a thermal decomposition treatment under preset conditions (Mercury/MA-3000 Mercury Analyser supplied by Nippon Instruments Corporation (NIC, Japan). MeHg was extracted by hydrolysis with hydrobromic acid (HBr) and then sequentially extracted with toluene and L-cysteine (Rutkowska et al. 2021). A free Social Science Statistics software (www.socscistatistics.com) and Microsoft Excel (2013 edition) were used for a basic statistical analyses of the results and including Mann–Whitney *U* test, and for illustrations.

## Results and discussion

The moisture content of the unprocessed carpophore in the various stages of development ranged from 90.0 to 90.1 % with a mean content at 90.0 ± 0.0%, while that of the braised products ranged from 73.1 to 76.5% with a mean of 74.8 ± 1.3% (Table 1). Individual data on THg and MeHg concentrations in culinary processed and unprocessed *B. edulis* for the different developmental stages and the net effect of braising (%) based on the whole (wet) and dry weight are detailed in Tables 2 and 3.

**Table 2:** Total mercury in stewed and crude *B. edulis* carpophore for developmental stages (mg kg^−1^ ww and mg kg^−1^ dw) and effect of stewing (%)

Developmental stage	Stewed	Crude	Effect^*^	Stewed	Crude	Effect^*^
	mg kg^−1^ ww	mg kg^−1^ ww	%	mg kg^−1^ dw	mg kg^−1^ dw	%
Button stage	0.5434 ± 0.0071	0.2929 ± 0.0030	+85	2.218 ± 0.029	2.929 ± 0.030	^−^24
Young—white	0.2668 ± 0.0090	0.2434 ± 0.0014	+9.6	1.038 ± 0.035	2.434 ± 0.014	^−^57
Large—white	0.2813 ± 0.0035	0.1880 ± 0.0247	+50	1.197 ± 0.015	1.899 ± 0.025	^−^37
Large—yellow	0.3502 ± 0.0023	0.2249 ± 0.0016	+56	1.302 ± 0.008	2.272 ± 0.016	^−^42
All	0.3604 ± 0.0915	0.2373 ± 0.0308	52 ± 31	1.439 ± 0.390	2.383 ± 0.298	40 ±14

Notes: ^*^Data not corrected for hidden fatty residue adsorbed by carpophore, what had a weak diluting effect

**Table 3:** Methylmercury in stewed and crude *B. edulis* carpophore for developmental stages (mg kg^−1^ ww and dw), effect of stewing (%), and contribution of MeHg in THg content (%)

Developmental stage	Stewed	Crude	Effect^*^	Stewed	MeHg in THg	Crude	MeHg in THg	Effect^*^
	mg kg^−1^ ww	mg kg^−1^ ww	%	mg kg^−1^ dw	%	mg kg^−1^ dw	%	%
Button stage	0.0089 ± 0.0001	0.0028 ± 0.0000	+218	0.0364 ± 0.0006	1.6	0.0285 ± 0.0003	1.0	+28
Young—white	0.0039 ± 0.0000	0.0043 ± 0.0007	^−^9.3	0.0152 ± 0.0001	1.5	0.0431 ± 0.0007	1.8	-65
Large—white	0.0023 ± 0.0016	0.0062 ± 0.0000	^−^63	0.0097 ± 0.0067	0.8	0.0622 ± 0.0005	3.3	-84
Large—yellow	0.0060 ± 0.0000	0.0036 ± 0.0000	+67	0.0223 ± 0.0002	1.7	0.0363 ± 0.0002	1.6	-39
All	0.0053 ± 0.0022	0.0042 ± 0.0010	+53 ± 122	0.0209 ± 0.0084	1.4 ± 0.3	0.0425 ± 0.0101	1.9 ± 0.7	-40 ± 49

Notes: ^*^Results not corrected for content of fatty residue adsorbed by carpophore, what had a weak diluting effect

### THg, braising, and carpophore developmental pattern

Total Hg concentration in braised carpophores in the whole (wet) weight for the developmental stages was in the range from 0.2668 ± 0.0090 to 0.5434 ± 0.0071 mg kg^−1^ whole (wet) weight (ww). The mean error for all maturity stages altogether was 0.3604 ± 0.0915 mg kg^−1^ ww. THg in the unprocessed (raw) carpophores ranged from 0.1880 ± 0.0247 to 0.2929 ± 0.0030 mg kg^−1^ ww with an overall mean of 0.2373 ± 0.0308 mg kg^−1^ ww (Table 2). Both raw and braised button stage carpophores were more contaminated with THg than those at higher maturity stages, on a dry weight as well as whole weight basis (*p* < 0.0001; M–W *U* test). It has to be pointed out that results for braised carpophores were not corrected for the amount of fat absorbed during braising which would have a slightly diluting effect. The tested fats (butter and olive oil) showed no detectable content of THg (method limit of detection was close to 0.001 mg kg^−1^ and method limit of quantification was close to 0.003 mg kg^−1^) (Rutkowska et al. 2021). The increase in THg concentration in braised carpophores can be explained by the partial decline in moisture content during braising and the resulting shrinkage of the fungal matrix accompanied by the preferential retention of a portion of THg. In other words, to obtain a portion of 100 g braised carpophores of *B. edulis*, a higher amount of fresh carpophores would be required. The braising process resulted in an increase in THg concentration of the mushroom meal products from 9.6 to 85% ww (mean: 52% ± 31% ww) across all the developmental stages studied.

The crude, dehydrated carpophores showed THg concentrations in the range from 1.899 ± 0.025 to 2.929 ± 0.030 mg kg^−1^ dw, across all developmental stages, with an overall mean of 2.383 ± 0.298 mg kg^−1^ dw (Table 2). If fully dehydrated fungal material weights are used as a basis to estimate the effect of braising, the reduction of THg content was 24% for the button stage group and from 37 to 57% for higher maturity stages (total by 40 ±14%). This would be expected as the THg content of the lyophilized raw *B. edulis* samples increased tenfold due to the removal of moisture.

The partial removal of Hg from a fungal substrate during the braising experiment is possible because of suspected co-distilling with water vapor and other volatiles through the hole in a glass lid. Further minor losses may also arise due to condensation (deposition) on the inner surface of the lid as well as absorption by the oily film and a small amount of residual fat remaining on the internal surface of the pot after transfer of the braised mushroom meal. An increase of Hg content in braised mushrooms if expressed on dry weight basis could only be possible if a fortification of the meal with Hg from an external source took place.

There is some comparative data on the Hg content in mushrooms at some developmental stages in the scientific literature. For example, Seeger (1976) noted that in *Agaricus campester* (current name *A. campestris* L.—many names and varieties for this mushroom are available from the Index Fungorum web site), *Agaricus silvicola* (current name *A*. *silvicolae-similis* Bohus and Locsmándi), and *B. edulis*, the “young mushrooms seemed to contain more Hg then older ones,” based on dry weight. Similarly, Nasr and Arp (2011) and Nasr et al. (2012) examined THg in carpophores of 27 species of macrofungi and noted a significant decline in THg (dry weight data) from emergence to senescence in 26 species, except, curiously, *B edulis*. For *B. edulis*, concentration of THg in carpophores increased from emergence to mature and to senescence developmental stage (Nasr and Arp 2011). It seems possible that the description of “young mushrooms” or “emergence” developmental stage in cited studies could be different from the “button stage” in the current study. Here, unlike Nasr et al. (2012), no such correlation has been observed; however, large fruiting bodies with yellow hymenophore were still perfect for consumption. In a study of *Amanita muscaria* (L.) Lam., the THg concentrations in carpophores were similar regardless of their developmental stage from the bottom size to fully mature but not at a “rotting” condition (six size classes) (Hanć et al. 2021).

### MeHg, braising, and carpophore developmental pattern

The overall mean MeHg concentration in braised meals across all the developmental stages was 0.0053 ± 0.0022 mg kg^−1^ ww, while that for the unprocessed (raw) mushrooms was 0.0042 ± 0.0010 mg kg^−1^ ww. The MeHg content of a braised meal made of the button stage carpophores was 0.0089 ± 0.0001 mg kg^−1^ ww but this concentration decreased in the higher developmental stages in which the overall mean was 0.0041 ± 0.0019 mg kg^−1^ ww (difference statistically significant; *p* < 0.0001; M–W *U* test). Braising resulted in increase of MeHg in mushroom meals by 53 ± 122% whole (wet) weight (a decrease by 40 ± 49% on dry weight basis) but these differences were variable between the different stages (Table 3). The raw dehydrated carpophores across all developmental stages showed MeHg at concentration of 0.0425 ± 0.0101 mg kg^−1^ dw with much lower concentrations in the braised counterparts, i.e., 0.0209 ± 0.0084 mg kg^−1^ dw.

The braised mushrooms in this experiment, as already mentioned, retained some amount of fatty residue (around 5 to 7% ww) which has a diluting effect on the basic constituents retained. Usually, apart from fat (butter or vegetable oil), recipes for braised mushrooms can also include a variety of vegetables and spices but also meat. However, the basic meal of *B. edulis* and butter or olive oil is adequate as a meal. Notably, domestic drying of fungal carpophores (traditionally, mushrooms are roughly sliced and dried in partial sunshine—a common technique to preserve edible wild mushrooms around the world), regardless of the manner, always leads to the pre-concentration at almost tenfold high rate of solids, i.e., the constituents that are “nonvolatile” at a given temperature regime.

The MeHg contribution to THg in crude and braised carpophores of *B. edulis* regardless of the developmental stage was low, i.e., at 1.9 ± 0.7% in crude and 1.4 ± 0.3% in braised product and did not show any tendency to increase or decrease during development (Table 3). In this study, the scale of the MeHg contribution to THg in raw *B. edulis* carpophores was evidently lower when compared to results from other studies described in the “Introduction” section. In a most recent study of composite samples of a dried caps, stipes and whole fruiting bodies of *B. edulis* from a several sites, the MeHg contribution to THg was in the range from 0.50 to 4.9% (Rutkowska et al. 2021). The scientific literature lacks data on MeHg for culinary processed mushrooms. At the button stage, *B. edulis* showed higher MeHg concentrations than crude sample, which might suggest better retention during braising, but this pattern was not seen for the higher developmental stages (Table 3).

For human consumption, the provisional tolerable weekly intake (PTWI) of inorganic Hg (iHg) is 0.004 mg kg^−1^ body mass (bm) and of MeHg is 0.0016 mg kg^−1^ bm (World Health Organization 2007), i.e., 0.280 mg of iHg and 0.112 mg of MeHg for a person of 70 kg bm per week. In this study, consumption of a 100 g meal of braised *B. edulis* once in a week would result in a total MeHg intake of 0.00053 ± 0.00022 mg, and 0.03604 ± 0.00915 mg of THg, which are both well below the PTWI.

Mushrooms are organisms that are capable of absorption and high bio-concentration of Hg, but at the same time, they are also relatively rich in selenium (Se), which is an antagonist of Hg. This is seen in many species, particularly from the family *Boletaceae* and including *B. edulis*. Thus, apart from a small and considered as safe in this study, for human consumers, the potential intakes of THg and MeHg from braised meals of *B. edulis* studied were small and considered safe, but the Se content could be considered as an additional parameter preventing adverse effects from both Hg compounds. However, this was not included in the present study.

## Data Availability

Not applicable.
